# Paracoccidioidomycosis mimicking mycetoma

**DOI:** 10.1590/0037-8682-0210-2024

**Published:** 2025-01-27

**Authors:** Beatriz Zimermano Coimbra, Bianca Tazinafi Lourenço, Marcelo Guimarães Tiezzi, Murilo de Oliveira Lima Carapeba, Marilda Aparecida Milanez Morgado de Abreu

**Affiliations:** 1 Universidade do Oeste Paulista, Programa de Pós-Graduação Stricto Sensu em Ciências da Saúde, Presidente Prudente, SP, Brasil.; 2 Hospital Regional de Presidente Prudente, Departamento de Dermatologia, Presidente Prudente, SP, Brasil.; 3 Universidade do Oeste Paulista, Faculdade de Medicina, Presidente Prudente, SP, Brasil.; 4 Hospital Regional de Presidente Prudente, Departamento de Patologia, Presidente Prudente, SP, Brasil.

**FIGURE 1:**
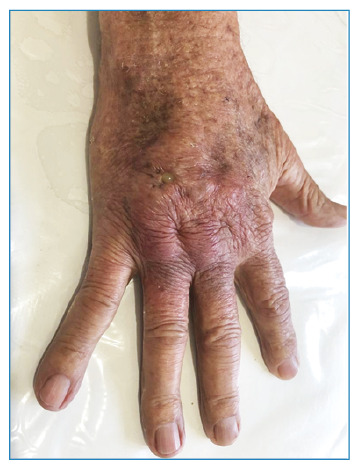
Erythematous nodule with hardened, retracted areas and floating areas with purulent exudate on the back of the right hand between the 2^nd^ and 4^th^ fingers.

**FIGURE 2:**
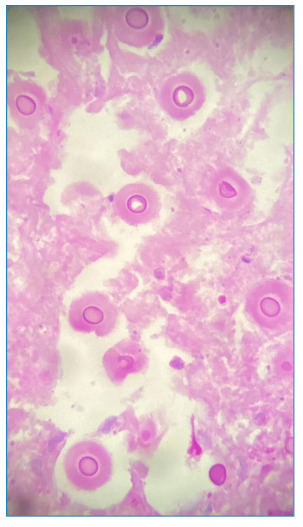
Histopathological examination: fungal spores compatible with *P. brasiliensis* stained by PAS - x400.

**FIGURE 3:**
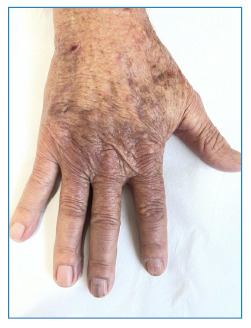
Total regression of the nodosity with residual hyperchromia.

Paracoccidioidomycosis (PCM) is a systemic mycosis endemic to Brazil, caused by fungi of the genus Paracoccidioides (*P. brasiliensis or P. lutzii*)^1^ ^-^ ^3^. Although there are different forms of fungal exposure, infections caused by agricultural activities are the most prevalent one^2^ ^,^ ^3^. We report the case of an 82-year-old man from the countryside of the state of São Paulo, who had an abscess and a fistulous lesion on his right hand experienced one year and five months earlier (Figure 1). The patient believed that the injury had arisen from a spider bite that he saw in his hand. The patient had been in agricultural work for more than 20 years, and during his free time, his hobby was gardening. Therefore, the clinical diagnosis of mycetoma was established. However, the pathological examination was compatible with PCM (Figure 2). Thus, the diagnosis of mycetoma-like paracoccidioidomycosis was conclusive. ‌ Itraconazole (200 mg/day) was administered. After nine months, the patient showed complete lesion regression (Figure 3). A plausible explanation for this case is that the organism reacted to the spider bite as trauma. Boni et al. reported that isolated lesions in patients with a history of previous trauma may correspond to the phenomenon called “locus minoris resistentiae,” in which the trauma “fixes” the fungus already in the fungemia phase^2^. To the best of our knowledge, this is the first reported case of paracoccidioidomycosis-mimicking mycetoma. Therefore, we highlighted the need for dermatologists to consider the presentation of PCM under non-suggestive conditions.
